# Impact of Preoperative Ocular Hypotony on Refractive Predictability After Phacovitrectomy for Rhegmatogenous Retinal Detachment

**DOI:** 10.3390/life16061009

**Published:** 2026-06-16

**Authors:** Ayana Kurobe, Kairat Ruslanuly, Sugao Miyagi, Fumito Akiyama, Akira Machida, Masafumi Uematsu, Akbala Dautaliyeva, Akio Oishi

**Affiliations:** 1 Graduate School of Biomedical Sciences, Nagasaki University, Nagasaki 852-8501, Japan; ayana0814k@gmail.com (A.K.); uematsu@nagasaki-u.ac.jp (M.U.); akio.oishi@nagasaki-u.ac.jp (A.O.); 2 Science Management Department, Kazakh Eye Research Institute, Almaty 050012, Kazakhstan; ruslanuly.kairat@gmail.com; 3 Department of Ophthalmology and Visual Sciences, Nagasaki University, Nagasaki 852-8501, Japan; miyagi@nagasaki-u.ac.jp (S.M.); fumito.akiyama@nagasaki-u.ac.jp (F.A.); a-machida@nagasaki-u.ac.jp (A.M.); 4 Department of Postgraduate Education, Kazakhstan Medical University “Higher School of Public Health”, Almaty 050060, Kazakhstan

**Keywords:** retinal detachment, ocular hypotension, refractive errors, vitrectomy, phacoemulsification

## Abstract

*Background/Objectives*: Rhegmatogenous retinal detachment is a vision-threatening condition often treated with combined cataract surgery and vitrectomy, but accurate postoperative refractive prediction may be difficult when ocular anatomy is altered by preoperative hypotony. This retrospective comparative study evaluated the effect of preoperative ocular hypotony on refractive predictability and visual outcomes after combined phacovitrectomy for rhegmatogenous retinal detachment. *Materials and Methods*: Electronic medical records from Nagasaki University Hospital from 2010 to 2022 were reviewed. Among 731 eyes that underwent combined phacovitrectomy with sulfur hexafluoride gas tamponade for rhegmatogenous retinal detachment, 17 eyes with preoperative ocular hypotony (intraocular pressure ≤ 7 mmHg) that met the inclusion criteria were identified. Thirty-four eyes with normal intraocular pressure were selected as controls in a 1:2 ratio. Refractive outcomes were assessed through spherical equivalent and mean absolute error, and visual outcomes were evaluated through best-corrected visual acuity at baseline and 6 months postoperatively. *Results*: Mean absolute error was significantly higher in eyes with hypotony than in normotonic eyes (1.46 ± 0.95 diopters vs. 0.70 ± 0.81 diopters; *p* = 0.002; mean difference, 0.76 D; 95% CI, 0.21–1.31; Hedges’ g = 0.87). Target postoperative refraction within ±0.5 diopters was achieved in 23.5% of hypotonic eyes and 50.0% of normotonic eyes (*p* = 0.07), the corresponding proportions within ±1.0 D were 35.3% and 76.5%, respectively (*p* = 0.006). Best-corrected visual acuity improved significantly in both groups, but final visual acuity at 6 months was worse in hypotonic eyes (0.35 ± 0.31 vs. 0.09 ± 0.17 logarithm of the minimum angle of resolution; *p* < 0.001). *Conclusions*: Preoperative ocular hypotony in rhegmatogenous retinal detachment was associated with greater refractive prediction error and worse visual outcomes after phacovitrectomy.

## 1. Introduction

Rhegmatogenous retinal detachment (RRD) is a serious ocular condition characterized by the separation of the retina from its underlying supportive structures, the retinal pigment epithelium and the choroid [1]. RRD can lead to permanent vision loss if not treated promptly. Common symptoms of RRD include sudden flashes of light, an increase in floaters, and the progressive peripheral scotoma.

Ocular hypotony, defined as abnormally low intraocular pressure (IOP), typically below 6–8 mmHg, is another critical condition that can arise from various causes, including RRD, trauma, inflammation, or surgical complications [2]. It is often associated with symptoms such as blurred vision, eye discomfort, and structural changes to the eye.

Ocular hypotony can complicate the management of RRD by compromising the structural integrity of the eye, thereby impeding successful retinal reattachment [3,4]. On the contrary, surgery for RRD may lead to ocular hypotony as a result of surgical trauma or postoperative complications. Correcting hypotony in cases of RRD can help maintain appropriate IOP, improve retinal reattachment rate, and prevent further complications associated with persistently low IOP [4].

RRD surgery can accelerate cataract development, while pre-existing cataracts may obstruct the surgeon’s view during RRD surgery, potentially affecting surgical outcomes [5]. Addressing both conditions simultaneously by performing phacovitrectomy, a combined surgery involving cataract removal and pars plana vitrectomy offers several advantages [6]. It improves surgical visualization, facilitates more effective retinal reattachment, and reduces the need for additional surgery. This combined approach not only minimizes the risk of complications but also enhances overall patient recovery and visual outcomes. However, hypotony, by its nature of altering the shape of the eye, may affect the measurement of axial length (AL) needed to determine the power of intraocular lens (IOL) [7].

The decision to conduct this research arose from the need to improve treatment outcomes for patients with RRD complicated by ocular hypotony. This study focuses on understanding how ocular hypotony impacts AL, which plays a main role in the accurate calculation of IOL power. Ocular hypotony can lead to a reduction in AL, potentially resulting in significant deviations from predicted postoperative refractive outcomes. By examining these deviations, we aim to assess the extent to which ocular hypotony affects IOL power calculations and to develop strategies for achieving more accurate IOL power determination in patients undergoing combined phacovitrectomy surgery.

## 2. Materials and Methods

### 2.1. Study Settings

A comprehensive retrospective analysis was conducted using medical records from 2010 to 2022 at Nagasaki University Hospital, Nagasaki, Japan. This study was approved by the Nagasaki University Hospital Clinical Research Ethics Committee (NUH CREC), №24071105-2 and conducted in accordance with the Declaration of Helsinki. The need for additional written informed consent was waived by the NUH CREC due to the retrospective nature of this study, the absence of patient-identifiable information in the published data, and the absence of any additional interventions or examinations. The study was announced on the NUH CREC homepage and subjects who did not wish to participate in the study were given the opportunity to opt out. The data were accessed through the hospital’s electronic medical record system and included all consecutive records of patients diagnosed with RRD who underwent cataract surgery combined with vitrectomy and gas tamponade.

To compare the outcomes, a control group was established, consisting of patients who underwent the same surgical procedure in the same year but had normal IOP. A ratio of 1:2 (hypotony cases to controls) was selected to ensure adequate statistical power for detecting differences between the groups.

### 2.2. Eligibility Criteria

Inclusion criteria: 1. patients diagnosed with RRD with hypotony; 2. RRD surgery involved cataract surgery with vitrectomy and gas tamponade; 3. intraocular pressure equal or less than 7 mmHg by Goldmann applanation tonometry.

Exclusion criteria: 1. RRD with normal or high IOP; 2. RRD surgery with silicone oil tamponade or combined with a scleral buckle; 3. coexisting conditions such as glaucoma, congenital anomalies of the eyeball, corneal opacities; 4. history of eye trauma, previous uveitis, diabetes, systemic autoimmune diseases, or prior intraocular surgery; 5. lack of available biometric data or incomplete medical records.

### 2.3. Preoperative and Postoperative Evaluations

Each patient underwent a complete ophthalmologic examination prior to surgery and during the follow-up period, which extended up to 6 months postoperatively. These examinations included: slit-lamp examination, measurement of uncorrected visual acuity (UCVA) and best-corrected visual acuity (BCVA), IOP measurements, dilated fundus examination with indirect ophthalmoscopy, keratometry and refraction measurements.

BCVA were measured using the Landolt ring chart and converted to the logarithm of the minimum angle of resolution (LogMAR) for numerical analysis.

IOP was initially measured using a non-contact tonometer (NT-530, Nidek, Japan). In cases where hypotony was detected or IOP could not be measured accurately via non-contact tonometry, Goldmann applanation tonometry (Haag-Streit Tonometers, Koeniz, Switzerland) was used to determine the true IOP.

Keratometry and refractive error were obtained using an auto ref/keratometer (ARK-530A, Nidek, Gamagori, Japan). According to our standard clinical protocol, at least five consecutive autorefraction and keratometry measurements were obtained. Readings affected by obvious outliers were repeated and the final values were based on the most reproducible measurements, generally within approximately 0.25 D for spherical equivalent (SE) and keratometric power. The mean absolute refractive error (MAE) was defined as the absolute difference between the predicted spherical equivalent and the actual postoperative SE, calculated as: MAE = |predicted SE − actual postoperative SE|. Mean absolute deviation (MAD) was the average absolute prediction error. Median absolute prediction error (MedAE) was the median of the absolute prediction errors. Formula performance index (FPI) was a unitless composite indicator of formula accuracy used to summarize performance across metrics; higher values indicated better performance.

AL was measured using both optical biometry (ZEISS IOLMaster 500, Oberkochen, Germany) and ultrasound A-scan biometry (AL-3000, Tomey, Nagoya, Japan), and the ALs obtained by both methods were compared to assess measurement reliability. According to the standard clinical protocol, at least five consecutive AL measurements were obtained. The measurements were accepted only when the acquisition showed a stable retinal peak, acceptable signal quality, and an adequate signal-to-noise ratio (SNR), without obvious noise-related artifacts or inconsistent outliers.

IOL power calculation was performed using the SRK/T formula. Optical AL was used when foveal attachment was observed, fixation was reliable, signal quality was acceptable, and the AL of the RRD eye was comparable to that of the fellow eye. When these conditions were not met, particularly in cases with detached posterior pole involvement, poor fixation, low signal quality, inability to identify a reliable retinal peak, or clinically implausible discrepancy between optical and ultrasound AL values, ultrasound A-scan AL was preferentially used. The type of IOL implanted varied according to hospital procurement agreements and lens availability.

Detachment height, exact circumferential extent of detachment, standardized proliferative vitreoretinopathy (PVR) grade, choroidal detachment, and OCT images were not uniformly available in the retrospective dataset and therefore were not included as quantitative covariates.

### 2.4. Surgical Technique

Surgeries were performed using the Alcon Constellation Vision System (Alcon Laboratories, Fort Worth, TX, USA).

#### 2.4.1. Cataract Surgery

Cataract surgery was performed using a standard technique. A 2.4 mm corneal or scleral tunnel incision was made at around 11 o’clock position. In our clinical practice, combined phacovitrectomy was preferred over vitrectomy alone in patients older than 50 years or in eyes with any pre-existing lens opacity, even if mild; cataract progression after vitrectomy is common and lens opacity may compromise intraoperative visualization.

#### 2.4.2. Pars Plana Vitrectomy

Pars plana vitrectomy was performed in all cases using 23- or 25-gauge systems. The vitreous was removed up to the vitreous base, and shaving of the vitreous base was performed around retinal breaks. Retinopexy was achieved using endolaser photocoagulation. After the vitrectomy ports were removed, a 30-gauge syringe was used to inject sulfur hexafluoride (SF_6_) gas as the tamponade agent. Postoperative care included the use of combined antibacterial and anti-inflammatory eye drops for one month.

### 2.5. Statistical Analysis

Statistical analysis was performed using GraphPad Prism 10 software (GraphPad Software Inc., San Diego, CA, USA). Data are presented as mean ± standard deviation (SD) unless otherwise indicated. Between-group comparisons of continuous variables were performed using the Mann–Whitney U test. Within-group paired comparisons were performed using the Wilcoxon signed-rank test. For longitudinal BCVA comparison across three time points, repeated measures one-way ANOVA was used. Categorical variables were compared using the chi-square test or Fisher’s exact test, as appropriate. A *p*-value of <0.05 was considered statistically significant. Confidence intervals (CI) and effect sizes were added for key refractive and visual outcomes. Hedges’ g was used for standardized between-group differences, rank-biserial correlation was used for nonparametric comparisons, and odds ratios (ORs) were calculated for categorical refractive accuracy thresholds. For categorical refractive accuracy outcomes, 95% CIs were calculated using the Wilson method. Exploratory multivariable linear regression was performed to assess whether the association between hypotony and MAE persisted after adjustment for preoperative SE, macular status, and RRD duration.

## 3. Results

A total of 731 medical records from patients diagnosed with RRD who underwent cataract surgery combined with vitrectomy and SF_6_ tamponade at NUH between 2010 and 2022 were reviewed. Among these, 24 patients (3.28%) were identified with hypotony. Seven of these patients (29.17%) experienced early postoperative retinal redetachment and were excluded from further analysis. Consequently, 17 patients (2.33%) with RRD and hypotony were included in the study.

A total of 51 eyes were analyzed: 17 eyes in the Hypotony group and 34 eyes in the Normotony group. Baseline characteristics are presented in Table 1. No significant differences were observed in age, sex distribution, high myopia, PVR, macula-off RRD cases, or time to surgery (*p* > 0.05). Postoperatively, hypotony was not observed in any of the patients included in the study. At the final 6-month follow-up, all eyes included in the analysis remained anatomically attached. No additional retinal reattachment procedures were performed during the follow-up period in the included eyes.

### 3.1. Visual Acuity

Changes in BCVA are summarized in Table 2. In the Hypotony group, the mean preoperative BCVA was 1.13 ± 0.69 LogMAR, improving to 0.72 ± 0.34 LogMAR on the 10th postoperative day and further to 0.35 ± 0.31 LogMAR at 6 months (F[2,16] = 13.87, *p* < 0.001). In the Normotony group, the mean preoperative BCVA was 0.79 ± 0.55 LogMAR, improving to 0.37 ± 0.37 LogMAR on the 10th postoperative day and 0.09 ± 0.17 LogMAR at 6 months (F[2,33] = 41.03, *p* < 0.001).

Both groups showed significant improvement over time, but final BCVA was consistently better in the Normotony group (*p* < 0.001 at all time points). The between-group difference in 6-month BCVA was 0.27 LogMAR (95% CI, 0.10–0.44; Hedges’ g = 1.15).

### 3.2. Ocular Biometry

No significant differences were found in preoperative AL between the Hypotony and Normotony groups based on both optical measurements (24.49 ± 2.29 mm and 24.88 ± 1.92 mm, respectively; *p* = 0.304) and A-scan measurements (25.46 ± 1.88 mm and 25.59 ± 1.44 mm, respectively; *p* = 0.688).

RRD eyes showed a shorter AL compared with fellow eyes in both the Hypotony group (24.49 ± 2.29 mm vs. 25.99 ± 2.01 mm; *p* = 0.004) and the Normotony group (24.88 ± 1.92 mm vs. 25.51 ± 1.39 mm; *p* = 0.029), with the difference being more significant in the Hypotony group.

No between-group or within-group differences were detected in anterior chamber depth, lens thickness, or central corneal thickness (all *p* > 0.05).

No statistically significant difference in anterior chamber (AC) depth was observed between the Hypotony and Normotony groups in both optical and A-scan measurements (*p* > 0.05). Similarly, no significant intergroup or intragroup differences were found in lens thickness (LT) or central corneal thickness (CCT) (*p* > 0.05).

### 3.3. Refractive Outcomes

In the Hypotony group, the mean SE at discharge was 3.87 ± 2.85 D, remaining relatively stable at 3.96 ± 2.55 D at the 6-month follow-up (*p* = 0.955). In the Normotony group, SE shifted from 2.79 ± 1.71 D at discharge to 2.59 ± 1.58 D at the 6-month follow-up (*p* = 0.937).

**Figure 1:**
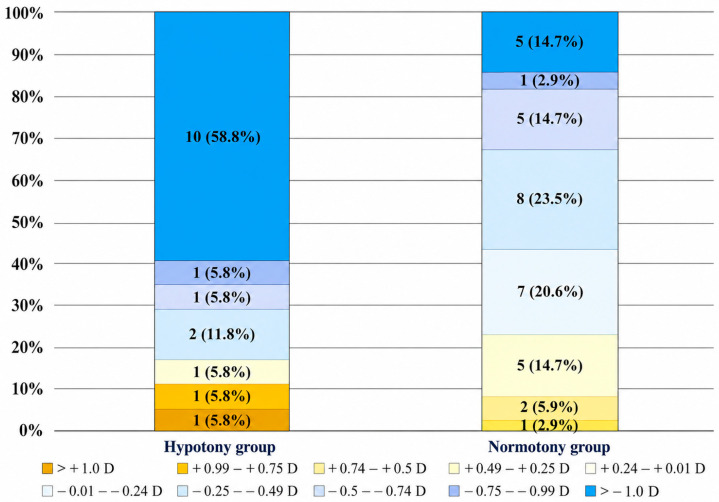
The stacked bar chart illustrates the distribution of residual refractive error (difference between the actual and target refraction, in diopters (D)) 6 months postoperatively in Hypotony and Normotony groups.

**Figure 2:**
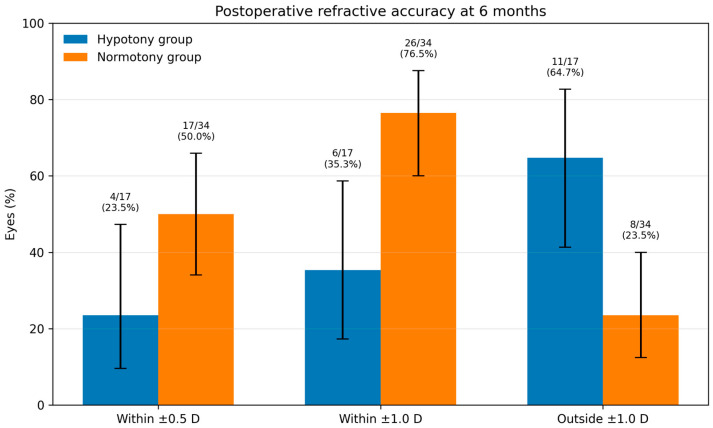
Proportion of eyes achieving postoperative refractive accuracy within ±0.5 D and ±1.0 D of target refraction and proportion of eyes outside ±1.0 D at 6 months after surgery. Error bars indicate 95% confidence intervals calculated using the Wilson method.

MAE was significantly higher in the Hypotony group (1.46 ± 0.95 D) compared to the Normotony group (0.7 ± 0.81 D, *p* = 0.002). The between-group difference was clinically meaningful (mean difference, 0.76 D; 95% CI, 0.21–1.31; Hedges’ g = 0.87; rank-biserial correlation = 0.47). The analysis of postoperative refraction at 6 months showed significant differences in refractive predictability between the groups (Figure 1). In the Hypotony group, only 23.5% of patients achieved target refraction (≤±0.5 D), whereas in Normotony group, it was achieved in 50.0% of cases (*p* = 0.07; Figure 2). The proportion of eyes within ±1.0 D was also lower in the Hypotony group (35.3% vs. 76.5%, *p* = 0.006, OR 0.17; 95% CI 0.05–0.60). Preoperative SE was not significantly correlated with MAE in the overall cohort (Spearman rho = −0.10, *p* = 0.474). In an exploratory adjusted model including hypotony status, preoperative SE, macular status, and RD duration, hypotony remained independently associated with higher MAE (β = 0.68 D, 95% CI 0.16–1.19, *p* = 0.011).

### 3.4. Predictive Accuracy of IOL Formulas

We analyzed the predictive accuracy of seven IOL power formulas. In the Normotony group, all formulas showed a small myopic systematic error (means from −0.51 to −0.77 D) with comparable accuracy (MAE 0.74–0.84 D; MedAE 0.29–0.44 D; Table 3). The proportions within ±0.50 D and ±1.00 D were 58–67% and 76–79%, respectively. By aggregate metrics, Hoffer Q (MedAE 0.29 D; FPI 0.49) and Barrett Universal II (MedAE 0.39 D; FPI 0.45) showed a slight edge, while Kane had a marginally higher MAE (0.84 D; FPI 0.44). These inter-formula differences were small and of limited clinical relevance.

In the Hypotony group, a significant myopic shift was observed across all formulas (means −1.07 to −1.59 D) with substantially poorer predictability (MAE 2.84–3.24 D; MedAE 1.91–2.62 D; Table 4). No eyes achieved ± 0.50 D with any formula and only 6.25–12.5% were within ±1.00 D. While SRK/T (MedAE 1.91 D) and Holladay 1 (2.09 D) trended slightly lower than others and Haigis was the highest (2.62 D). Inter-formula ranking was inconsistent and clinically overshadowed by the large between-group effect. Therefore, these findings should not be interpreted as evidence of the superiority of any specific IOL calculation formula in hypotonic RRD eyes, but rather as exploratory and descriptive observations.

## 4. Discussion

Hypotony is a well-recognized complication of RRD. Studies indicate that the prevalence of hypotony before surgery varies widely from 7.5% to 35.7%, depending on the extent of detachment, duration of detachment, and the presence of additional pathological factors [8,9]. In our study, the incidence of hypotony was 3.28% which may be explained by early intervention after the onset of the symptom (5 days on median).

Ocular hypotony in RRD may occur due to increased aqueous outflow and reduced aqueous production, leading to progressive IOP decline [10,11]. As the retina detaches, vitreous fluid enters the subretinal space and drains through the RPE pump, peri-optic connective tissue, or choroidal circulation, bypassing the trabecular meshwork and causing fluid loss. Ciliary body dysfunction from edema, detachment, or fibrosis further reduces aqueous production, while increased uveoscleral outflow and choroidal vessel leakage promote hypotony. The IOP may decrease substantially when the retinal detachment involves about half of the retinal area and/or reaches the optic disk; however, published data are heterogeneous, and the relationship is not strictly linear [10]. Further extension of detachment does not significantly decrease IOP. Hypotony accelerates the breakdown of the blood–ocular barrier, triggering inflammatory mediator release and retinal pigment epithelium migration, leading to worse surgical outcomes [12].

This study analyzed the impact of preoperative hypotony on refractive outcomes, ocular biometry, and visual acuity recovery in patients after phacovitrectomy with SF_6_ tamponade for RRD. We found that patients with hypotony had significantly greater refractive variability, as reflected by a higher MAE (1.46 ± 0.95 D) and a lower proportion of cases achieving the target refraction ≤ ±0.5 D (23.5%) compared to the Normotony group (0.70 ± 0.81 D and 50.0%, respectively). Despite both groups experiencing significant improvements in BCVA, final visual acuity outcomes were superior in the Normotony group (0.03 ± 0.12 compared to 0.35 ± 0.31 in the Hypotony group). However, this finding should not be interpreted as evidence that hypotony alone directly caused poorer visual recovery. Preoperative hypotony did not occur in all eyes with RRD, but only in a subset of cases, suggesting that its presence may reflect greater ocular structural instability or more advanced underlying retinal pathology. Therefore, hypotony should be considered not only as an isolated intraocular pressure finding, but also as a possible clinical marker of more severe disease. Because several retinal disease-severity variables could not be uniformly assessed in this retrospective cohort, causality cannot be inferred, and this analysis should be considered exploratory and descriptive.

Shi et al. revealed that postoperative refractive shift may be explained by change in AL by ≥0.3 mm in 43.4% and risk of increase is 2.77-fold higher than that of patients without hypotony [9]. They calculated that in hypotonic or in extremely myopic eyes, AL had an average increase of 0.46 mm, approximately 1.15 D myopia shift. This research may explain our prevailed myopic shift by more than 1 D in 58.8% of cases.

A study conducted by Yu et al. and Adelman et al. demonstrated that biometric changes significantly influence the development of ocular hypotony, a highly complex process requiring the simultaneous presence of multiple contributing factors [13,14]. These factors include posteriorly located retinal breaks, particularly in cases of macular hole-associated RRD, a larger area of retinal detachment, and a longer AL. BCVA prognosis following RRD surgery is heavily influenced by macular involvement and the duration of detachment. The Japan-Retinal Detachment Registry reported that macula-off RRDs were observed in 20.7% of patients and visual acuity significantly worsened with prolonged macular detachment, with the best outcomes observed when surgery was performed within 3 days [15]. Similarly, Lee et al. reported that final visual acuity in macula-off RRD patients (LogMAR 0.04 ± 0.07) with detachment duration ≤ 3 days was comparable to that of macula-on RRD (LogMAR 0.05 ± 0.06, *p* = 0.79), whereas delays beyond 4–7 days resulted in significantly worse visual outcomes (LogMAR 0.36 ± 0.29, *p* < 0.001 compared to macula on) [16]. In the context of our study, despite significant postoperative improvement in both groups, the final visual acuity remained worse in the Hypotony group (0.35 ± 0.31 LogMAR) compared to the Normotony group (0.03 ± 0.12 LogMAR, *p* < 0.001). These findings support prior evidence that hypotony-associated RRD is linked to worse visual recovery due to greater structural damage at baseline.

Accurate IOL power calculation is a crucial factor in achieving optimal refractive outcomes following phacovitrectomy for RRD. However, multiple factors can contribute to refractive prediction errors, leading to unexpected hyperopic or myopic shifts postoperatively. In our study, refractive outcomes demonstrated significant variability in the Hypotony group, with a greater MAE and a >2.0 D increase in mean absolute prediction error across all modern IOL power formulas compared to the Normotony group, indicating that accurate IOL power calculation remains a challenge in RRD cases with hypotony, where macula-off detachment was observed in nearly all cases. Nevertheless, because the formula comparison was underpowered, the formula-specific findings should be interpreted as exploratory and should not guide formula selection independently of clinical judgment.

Findings from El-Khayat et al. further support this observation, showing that in macula-off RRD, using same-eye biometry resulted in a significantly greater MAE of 1.57 ± 2.08 D, with a notable myopic shift (mean prediction error −1.22 D), compared to fellow-eye biometry, which produced a lower MAE of 0.73 ± 0.80 D [17]. They reported that 34.1% of eyes achieved an absolute prediction error within 0.5 D. In comparison, our study demonstrated even poorer refractive predictability in hypotonic eyes, with only 23.5% of patients in the Hypotony group achieving target refraction (≤±0.5 D), whereas in the Normotony group, it was achieved in 50.0% of cases. Considering that El-Khayat et al. examined only macula-off RRD, the addition of hypotony in our cohort likely exacerbated the calculation errors. Although 50.0% of normotonic eyes were within ±0.5 D of target refraction, which is modest compared with contemporary uncomplicated cataract surgery, this level of accuracy is not unexpected in the setting of phacovitrectomy for RRD. Unlike routine cataract surgery, RRD phacovitrectomy is performed in eyes with altered posterior segment anatomy. Previous studies have similarly reported lower refractive predictability after phacovitrectomy for RRD than after phacovitrectomy for non-RRD indications. Shiraki et al. reported that 44% and 68% of RRD eyes were within ±0.50 D and ±1.00 D, respectively [18]. In this context, the 50.0% within ±0.5 D and 76.5% within ±1.0 D observed in our normotony group appear broadly consistent with the expected refractive limitations of RRD phacovitrectomy.

We hypothesize that in hypotonic eyes all relevant dimensions shift by very small amounts. These mechanisms were not directly measured in the present study and should therefore be regarded as biologically plausible explanations rather than proven causal pathways. Hypotony may lead to shallowing of the anterior chamber, choroidal expansion, and altered capsular bag-zonular tension. Formulas calibrated on normotensive eyes may under- or mis-estimate the effective lens position (ELP) and a more anterior postoperative IOL position than predicted produces a myopic outcome. Even subtle steepening of cornea during preoperative biometry can propagate into a systematic myopic error after surgery when IOP normalizes. Retinal reattachment and IOP normalization can increase the effective optical path length compared with measurements obtained in a hypotonic, detached state. Cumulatively, these sub-detectable shifts can sum to a clinically meaningful refractive error, and these mechanisms all act in the same direction (anterior ELP and/or longer effective optics postoperatively), which matches the uniform myopic mean prediction errors we observed in the Hypotony group across formulas. Prospective, adequately powered studies with standardized timing and high-precision ocular biometry measures, ELP, keratometry repeatability, choroidal thickness, and postoperative IOL position are needed to confirm this mechanism. These findings suggest that same-eye biometry in hypotonic RRD eyes is particularly prone to inaccuracies, highlighting the need for alternative strategies, such as fellow-eye biometry or adjusted AL measurements, to improve refractive predictability.

This study has several limitations. First, the sample size, especially in the Hypotony group, is small, which reduces precision and limits the power of statistics. Second, the retrospective, single-center design is susceptible to selection and information bias. Notably, we excluded other seven hypotonic eyes from the analyses; although reasons for exclusion were predefined, this attrition may introduce selection bias. Third, our cohort only included cases managed with gas tamponade; eyes requiring silicone oil, scleral buckling, or combined approaches were excluded, so generalizability is limited to gas tamponade phacovitrectomies for RRD. Fourth, biometry methods and IOL constants were not fully standardized over the study period, and the timing of measurements relative to IOP normalization and macula status varied, which may have added measurement noise. Postoperative AL was not routinely reassessed; therefore, the proposed normalization of AL after retinal reattachment remains an explanatory hypothesis rather than a directly measured outcome. Fifth, the formulas were evaluated on the same eyes, errors are correlated rather than independent, and our small sample was not powered for definitive inter-formula ranking; therefore, comparisons between formulas should be interpreted with caution. Sixth, several potentially important retinal confounders, including detachment height, exact detachment extent, standardized PVR grade, choroidal detachment, and detailed OCT-based macular morphology, were not uniformly available and could not be included in the adjusted analyses. Finally, we did not acquire high-precision metrics such as intraoperative aberrometry, anterior chamber/ELP, or corneal biomechanical indices, which could clarify mechanisms and refine predictive models. Prospective, adequately powered studies with standardized biometry timing and detailed anterior/posterior segment metrics are warranted.

## 5. Conclusions

Our study highlights the significant impact of preoperative hypotony on refractive predictability following phacovitrectomy for RRD. The greater refractive variability, increased MAE, and reduced likelihood of achieving target refraction in the Hypotony group highlight the need for meticulous biometric adjustments in these patients. Given the retrospective design, limited hypotony cohort, and incomplete availability of retinal severity variables, these findings should be interpreted as exploratory and hypothesis-generating. Further research is required to refine IOL power calculation strategies and improve visual outcomes in eyes affected by hypotony-associated RRD.

## Figures and Tables

**Table 1:** Baseline characteristics of patients.

Parameters	Hypotony Group	Normotony Group	*p*-Value
Eyes (n)	17	34	na
Male/female (%)	10/7 (59.4)	23/11 (67.7)	0.534 ^1^
Mean age ± SD (years)	58.0 ± 8.4	58.7 ± 8.5	0.779 ^2^
Preoperative RRD eye SE (D)	−5.2 ± 4.36	−3.86 ± 3.59	0.248
Macula-on/off RRD (%)	4/13 (23.5)	13/21 (38.2)	0.294 ^1^
High myopia ≥ 26.0 mm, n (%)	6 (35.3)	14 (41.2)	0.685 ^1^
PVR, n (%)	14 (82.4)	26 (76.5)	0.63 ^1^
Days from RRD event to surgery (Median, IQR)	5, 1.5–9.5	7, 3–19.25	0.141 ^3^

na—not applicable, SD—standard deviation, PVR—proliferative vitreoretinopathy, RRD—rhegmatogenous retinal detachment, IQR—interquartile range, SE—spherical equivalent. ^1^ chi-square test, ^2^ parametric unpaired *t* test, ^3^ nonparametric Mann–Whitney test.

**Table 2:** Changes in best-corrected visual acuity (BCVA) in logarithm of the minimum angle of resolution (LogMAR) in Hypotony and Normotony groups.

Parameters	Preoperative	10th Postoperative Days	6 Months Postoperative	*p*-Value ^1^	df, F
Hypotony group	1.13 ± 0.69	0.72 ± 0.34	0.35 ± 0.31	<0.001 *	F(2, 16) = 13.87
Normotony group	0.79 ± 0.55	0.37 ± 0.37	0.09 ± 0.17	<0.001 *	F(2, 33) = 41.03
*p*-value ^2^	0.052	<0.001 *	<0.001 *		

df—degrees of freedom, F—F-statistic value, * statistical significance, ^1^ repeated measures one-way ANOVA, ^2^ nonparametric Mann–Whitney test.

**Table 3:** Overall predictive accuracy of IOL calculation formulas for Normotony group.

Formula	Mean	SD	MAE	MedAE	FPI	Percentage of Eyes Within PE (%)			
						±0.25 D	±0.50 D	±0.75 D	±1.00 D
Hoffer Q	−0.51	1.21	0.74	0.29	0.49	48.48%	66.67%	75.76%	75.76%
Holladay 1	−0.53	1.22	0.80	0.40	0.47	36.36%	57.58%	75.76%	78.79%
SRK/T	−0.66	1.19	0.81	0.39	0.46	27.27%	60.61%	72.73%	78.79%
Haigis	−0.56	1.28	0.83	0.42	0.45	24.24%	66.67%	75.76%	78.79%
BUII	−0.68	1.22	0.81	0.39	0.45	39.39%	66.67%	75.76%	78.79%
EVO	−0.75	1.19	0.81	0.43	0.46	38.71%	61.29%	74.19%	74.19%
Kane	−0.77	1.18	0.84	0.42	0.44	42.42%	60.6%	75.76%	75.76%

BUII = Barrett Universal II; D = diopter; FPI = formula performance index; MAE = mean absolute prediction error; MedAE = median absolute prediction error; PE = prediction error; SD = standard deviation.

**Table 4:** Overall predictive accuracy of IOL calculation formulas for Hypotony group.

Formula	Mean	SD	MAE	MedAE	FPI	Percentage of Eyes Within PE (%)			
						±0.25 D	±0.50 D	±0.75 D	±1.00 D
Hoffer Q	−1.54	3.23	2.99	2.51	0.182	0.0%	0.0%	6.25%	6.25%
Holladay 1	−1.53	3.26	2.9	2.09	0.2	0.0%	0.0%	6.25%	12.5%
SRK/T	−1.39	3.11	2.79	1.91	0.213	0.0%	0.0%	6.25%	6.25%
Haigis	−1.59	3.45	3.24	2.62	0.17	0.0%	0.0%	0.0%	12.5%
BUII	−1.39	3.16	2.95	2.3	0.191	0.0%	0.0%	6.25%	6.25%
EVO	−1.07	2.77	2.84	2.22	0.198	0.0%	0.0%	6.25%	6.25%
Kane	−1.32	3.13	2.87	2.28	0.194	0.0%	0.0%	6.25%	6.25%

BUII = Barrett Universal II; D = diopter; FPI = formula performance index; MAE = mean absolute prediction error; MedAE = median absolute prediction error; PE = prediction error; SD = standard deviation.

## Data Availability

The datasets of the current study are available from Ayana Kurobe upon reasonable request.

## Abbreviations

AC	Anterior chamber
AL	Axial length
ANOVA	Analysis of variance
BCVA	Best-corrected visual acuity
BUII	Barrett Universal II
CCT	Central corneal thickness
CI	Confidence intervals
CREC	Clinical Research Ethics Committee
D	Diopter
ELP	Effective lens position
EVO	Emmetropia Verifying Optical formula
FPI	Formula performance index
IOL	Intraocular lens
IOP	Intraocular pressure
IQR	Interquartile range
LogMAR	Logarithm of the minimum angle of resolution
LT	Lens thickness
MAE	Mean absolute prediction error
MedAE	Median absolute prediction error
Min	Minimum
NUH	Nagasaki University Hospital
OR	Odds ratio
PE	Prediction error
PVR	Proliferative vitreoretinopathy
RPE	Retinal pigment epithelium
RRD	Rhegmatogenous retinal detachment
SD	Standard deviation
SE	Spherical equivalent
SF_6_	Sulfur hexafluoride
SRK/T	Sanders–Retzlaff–Kraff/Theoretical formula
UCVA	Uncorrected visual acuity

## References

[1] Polkinghorne P.J., Craig J.P. (2004). Analysis of symptoms associated with rhegmatogenous retinal detachments. Clin. Exp. Ophthalmol..

[2] Abbas A., Agrawal P., King A.J. (2018). Exploring literature-based definitions of hypotony following glaucoma filtration surgery and the impact on clinical outcomes. Acta Ophthalmol..

[3] Tokuç E., Karabaş V.L., Seyyar S.A., Emengen E.B., Güray A.B., Dinçer K.A., Önder C.D., Özgür E.G. (2024). A novel predictor of persistent ocular hypotony after pars plana vitrectomy for rhegmatogenous retinal detachment: The initial intraocular pressure difference between the eye with RRD and the fellow eye. Arq. Bras. Oftalmol..

[4] Gkizis I., Garnavou-Xirou C., Bontzos G., Smoustopoulos G., Xirou T. (2021). Hypotony Following Intravitreal Silicone Oil Removal in a Patient with a Complex Retinal Detachment with Giant Retinal Tear. Cureus.

[5] Bellucci C., Benatti L., Rossi M., Tedesco S.A., Carta A., Calzetti G., Gandolfi S., Mora P. (2022). Cataract progression following lens-sparing pars plana vitrectomy for rhegmatogenous retinal detachment. Sci. Rep..

[6] Villegas V.M., Gold A.S., Latiff A., Wildner A.C., Ehlies F.J., Murray T.G. (2014). Phacovitrectomy. Dev. Ophthalmol..

[7] Matsumoto Y., Fujihara M., Kanamori A., Yamada Y., Nakamura M. (2014). Effect of axial length reduction after trabeculectomy on the development of hypotony maculopathy. Jpn. J. Ophthalmol..

[8] Adelman R.A., Parnes A.J., Sipperley J.O., Ducournau D. (2013). Strategy for the management of complex retinal detachments: The European vitreo-retinal society retinal detachment study report 2. Ophthalmology.

[9] Shi J., Wu K., Wen H., Wei J., Zong Y., Yu J., Zhu H., Jiang C. (2022). Change in axial length after vitrectomy with silicone oil tamponade for rhegmatogenous retinal detachment. BMC Ophthalmol..

[10] Solberg T., Ytrehus T., Ringvold A. (1986). Hypotony and retinal detachment. Acta Ophthalmol..

[11] Dai Y., Wu Z., Sheng H., Zhang Z., Yu M., Zhang Q. (2015). Identification of inflammatory mediators in patients with rhegmatogenous retinal detachment associated with choroidal detachment. Mol. Vis..

[12] Kwon O.W., Song J.H., Roh M.I. (2016). Retinal Detachment and Proliferative Vitreoretinopathy. Dev. Ophthalmol..

[13] Yu Y., An M., Mo B., Yang Z., Liu W. (2016). Risk factors for choroidal detachment following rhegmatogenous retinal detachment in a chinese population. BMC Ophthalmol..

[14] Adelman R.A., Parnes A.J., Michalewska Z., Ducournau D. (2014). Clinical variables associated with failure of retinal detachment repair: The European vitreo-retinal society retinal detachment study report number 4. Ophthalmology.

[15] Miyake M., Nakao S.Y., Morino K., Yasukura S., Mori Y., Ishihara K., Muraoka Y., Miyata M., Tamura H., Sakamoto T. (2023). Effect of Duration of Macular Detachment on Visual Prognosis after Surgery for Macula-Off Retinal Detachment: Japan-Retinal Detachment Registry. Ophthalmol. Retin..

[16] Lee C.S., Shaver K., Yun S.H., Kim D., Wen S., Ghorayeb G. (2021). Comparison of the visual outcome between macula-on and macula-off rhegmatogenous retinal detachment based on the duration of macular detachment. BMJ Open Ophthalmol..

[17] El-Khayat A.R., Brent A.J., Peart S.A.M., Chaudhuri P.R. (2019). Accuracy of intraocular lens calculations based on fellow-eye biometry for phacovitrectomy for macula-off rhegmatogenous retinal detachments. Eye.

[18] Shiraki N., Wakabayashi T., Sakaguchi H., Nishida K. (2018). Optical Biometry-Based Intraocular Lens Calculation and Refractive Outcomes after Phacovitrectomy for Rhegmatogenous Retinal Detachment and Epiretinal Membrane. Sci. Rep..

